# Analysis of work-related accidents and ill-health in Brazil since the introduction of the accident prevention factor

**DOI:** 10.1186/s12889-021-10706-y

**Published:** 2021-04-14

**Authors:** Helena Eri Shimizu, Josierton Cruz Bezerra, Luciano José Arantes, Edgar Merchán-Hamann, Walter Ramalho

**Affiliations:** 1 Universidade de Brasília, Faculdade de Ciências da Saúde, Departamento de Saúde Coletiva, Campus Universitário Darcy Ribeiro, Distrito Federal, Brasília, DF CEP 70910-900 Brazil; 2 Potiguar University, Av. Senador Salgado Filho, 1610, Lagoa Nova, Natal-RN, Natal, Estado do Rio Grande do Norte CEP 59056-000 Brazil; 3 Unaí Municipal Department of Health, Secretaria Municipal da Saúde de Unaí, Rua Calixto Martins de Melo, 249, Unaí, Minas Gerais CEP 38610-039 Brazil

**Keywords:** Occupational accidents, Occupational health, Occupational injuries, Social security

## Abstract

**Background:**

Since 2004, Brazil has had a national policy for occupational health and safety. This policy means companies’ tax burden is altered according to the numbers of work-related accidents and ill-health amongst their workers. In 2010, a multiplication factor was introduced to this policy, called the Accident Prevention Factor. The idea of this new multiplication factor is to encourage individual employers to take initiatives to prevent accidents and ill health in the workplace. This study was designed to investigate the incidence of work-related accidents and ill-health in Brazil according to their causes, their severity, and the economic activity in which they occur, and to compare the data before and after the introduction of the Accident Prevention Factor.

**Methods:**

An ecological study was conducted by analyzing the time series of work-related accidents/ill-health between 2008 and 2014 from the Brazilian social security system (Previdência Social) statistical yearbooks. Incidences were calculated per cause, economic activity, and severity of the accident/ill-health. Data from before and after the introduction of the Accident Prevention Factor were compared using the Mann-Whitney test per cause and per economic activity. Statistical analyses were made using the SPSS software, with significance set at 5%.

**Results:**

A reduction in the incidence of work-related accidents/ill-health was found across all the groups of causes analyzed, except for the groups “external causes of morbidity and mortality” and “factors influencing health status and contact with health services.” Greater reductions were found for diseases of the musculoskeletal system and connective tissue and diseases of the nervous system. Reductions in work-related accidents/ill-health were found in the different economic activities and in the different severity groups. The highest reduction after the introduction of the Accident Prevention Factor was in manufacturing and production (*p* < 0.05).

**Conclusions:**

Overall, the incidence of accidents/ill-health was found to be on decline, except those with external causes of morbidity and mortality and those involving factors influencing health status and contact with health services. The biggest reduction was found in manufacturing and production. However, generally speaking progress still needs to be made in accident prevention and occupational health across a whole range of work environments.

**Supplementary Information:**

The online version contains supplementary material available at 10.1186/s12889-021-10706-y.

## Background

Work is important for enhancing self-esteem, wellbeing, and social mobility [1]. However, accidents caused by or occurring during work activities are a major cause of impairments to workers’ health and have serious social and economic repercussions [2].

Internationally, the cost of work-related accidents and ill-health is estimated at between 1.8 and 6.0% of gross domestic product [3]. Every year, around 2.3 million people die around the world from work-related causes. In Brazil, there are about 2500 such deaths a year, which corresponds to one death every 3.5 h [4].

According to the Brazilian social security department (Secretaria de Previdência Social), the country spent around 330 million reais (101.5 million dollars) in 2016 on work-related accidents/ill-health. This figure relates to the social security payments alone, excluding associated healthcare costs, which would bring the figure even higher.

Since 2004, Brazil has had a national policy for occupational health and safety (Política Nacional de Segurança e Saúde do Trabalhador) [5]. Developed after the passing of law 10.666 on May 8, 2003, this policy means companies’ tax burden is altered according to the numbers of work-related accidents and ill-health amongst their workers. It is designed to levy extra taxes from companies where occupational accidents/ill-health occur, making them contribute towards the social security payments made to the affected workers [5].

In Brazil, corporate tax is levied according to the incidence of injuries that take place across each economic sector. Companies are therefore allocated to one of three brackets according to the risk factor inherent to their line of business: 1% for low-risk activities, 2% for medium-risk activities, and 3% for high-risk activities. In 2010, a multiplication factor was introduced to this policy, called the Accident Prevention Factor. Essentially, the risk factors at which companies are taxed, as described above (1, 2% or 3%), are multiplied by this new factor, which varies according to the frequency of the accidents/ill-health actually recorded by a company, ranging from 0.5 to 2.0 [5].

The idea of this new multiplication factor is to encourage individual employers to take initiatives to prevent accidents and ill health in the workplace. It aims to reduce accident rates according to the rationale of “bonus malus,” whereby employers responsible for more accidents/ill-health are penalized by paying higher taxes than other companies in the same line of business whose accident/ill-health rates are lower. It is worth noting that in Brazil, the term “work-related accident” encompasses both preventable and non-preventable accidents.

Researchers have analyzed the sociodemographic, economic, and cultural aspects of work-related accidents and ill-health [6–9]. However, little has been done to analyze the effects of policies designed to prevent them [10].

In view of the need to understand in greater depth the scenario of work-related accidents/ill-health in Brazil since the introduction of the Accident Prevention Factor and to help improve policies related to occupational health and safety, the aim of this study was to ascertain the incidences in work-related accidents/ill-health in Brazil from 2008 to 2014, looking at their causes, their severity, and the economic sectors in which they occur, while also comparing the data on incidence rates before and after the introduction of the Accident Prevention Factor.

## Methods

An ecological study was carried out using a time series from 2008 to 2014 with data from the Brazilian government’s social security platform (https://www3.dataprev.gov.br/aeat/) [11] and statistical yearbooks (https://www.previdencia.gov.br/dados-abertos/sst/) [12]. Data on work-related accidents and ill-health suffered by workers covered by the social security system were collected on October 6, 2016. The frequency of such cases was analyzed according to their causes, their severity, and the economic activities involved.

To analyze the causes of work-related accidents, the data were classified according to the International Statistical Classification of Diseases and Related Health Problems (ICD-10). In particular, we present here the data from the chapters for which the highest prevalence of work-related accidents were found: Injury, poisoning and certain other consequences of external causes (Chapter XIX); Diseases of the musculoskeletal system and connective tissue (Chapter XIII); External causes of morbidity and mortality (Chapter XX); Mental and behavioral disorders (Chapter V); Factors influencing health status and contact with health services (Chapter XXI); and Diseases of the nervous system (Chapter VI). Each chapter of ICD-10 was considered an analysis group. Data on the five causes with the highest prevalence per chapter were identified, and the most prevalent ones in each chapter were referred to as specific causes.

To study the economic activities undertaken by the workers, the data were grouped according to Brazil’s national classification of economic activities (Classificação Nacional de Atividades Econômicas, CNAE 2.0), again identifying the groups with the highest prevalence of work-related accidents/ill-health. According to the classification used (CNAE 2.0), an economic activity is understood as being any activity performed by a public or private enterprise that results in the production of goods and/or services. The economic activity groups with the highest prevalence of work-related accidents were: manufacturing and production; retail and motor vehicle repair; human health and social services; civil construction; and transportation, storage, and mail services. The five economic activities with the highest prevalence in each group were also identified.

Finally, to classify severity, the categories used were injury/illness for which medical attention was required, injury/illness resulting in less than 15 days off work, injury/illness resulting in more than 15 days off work, permanent disability, and death.

The incidence rates for each group were calculated by the number of new cases of work-related accidents/ill-health divided by the number of workers covered by the social security system multiplied by 1000. The incidence rates of each group of analysis at the beginning and end of the time series were compared. Also, the accumulated incidence rates were calculated by dividing the total number of accidents/ill-health by the total number of workers covered by the social security system in the same period. The calculations were done for the periods before and after the introduction of the Accident Prevention Factor.

The incidence rates of work-related accidents/ill-health before (2008 and 2009) and after (2011 to 2014) the introduction of the Accident Prevention Factor were compared using the Mann-Whitney test per cause and per economic activity. This test was used because the data were found not to have normal distribution, according to the Kolmogorov-Smirnov test (*p* > 0.05), as the comparison was done considering just the five most prevalent causes and occupations in each group (small *n*) and the quantity of data was different in the period before (*n* = 10) and after (*n* = 20) the introduction of the policy. The analyses were conducted using the Statistical Package for the Social Science (SPSS), version 22, with significance set at 5%. The severity of the accidents was not analyzed statistically because of the small quantity of data and its limited representativeness.

As this study used secondary data publicly available on the internet, it was not submitted for approval by the research ethics committee.

## Results

### Causes of work-related accidents

In the analysis of the causes of work-related accidents, a reduction was observed in the incidence of injury, poisoning and certain other consequences of external causes; diseases of the musculoskeletal system and connective tissue; mental and behavioral disorders; and diseases of the nervous system (Table 1). However, the incidence of cases involving external causes of morbidity and mortality and factors influencing health status and contact with health services rose (Table 1 and Additional file 1: Table S1).

**Table 1:** Incidence of work-related accidents/ill-health in Brazil using the ICD-10 (per 1000 workers)

Causes of accidents/ill-health	Difference in incidence between 2008 and 2014 (%)	Accumulated Incidence 2008–2009	Accumulated Incidence 2011–2014	Ratio of Accumulated Incidence	Mann-Whitney Test ***p***
Injury, poisoning and certain other consequences of external causes	−22.93	0.0128	0.0106	0.8309	0.40
-- injury to the wrist or hand	−33.42				
-- fracture at wrist or hand level	−22.43				
-- contusion of wrist or hand	−25.55				
-- dislocation or strain or sprain of joints or ligaments at ankle and foot level	−6.97				
-- fracture of leg, including ankle	−9.83				
Diseases of the musculoskeletal system and connective tissue	−43.64	0.0033	0.0022	0.6575	0.17
-- back pain	− 53.21				
-- shoulder lesions	− 29.21				
-- synovitis and tenosynovitis	− 60.83				
-- other intervertebral disc injury	− 32.90				
-- other joint derangements	−1.86				
External causes of morbidity and mortality	6.04	0.4467	0.4575	1.0240	0.95
-- bitten or struck by dog	10.40				
-- unintentional cut, puncture, perforation, or hemorrhage during medical or surgical procedure	−17.60				
-- unspecified harm during medical or surgical procedure	29.47				
-- cut or piercing with a sharp object, intentionality not determined	23.34				
-- assault by being cut or pierced	−46.50				
Mental and behavioral disorders	− 13.32	0.0004	0.0003	0.8621	0.78
-- reactions to severe stress and adjustment disorders	3.01				
-- depressive episodes	−43.96				
-- other anxiety disorders	35.05				
-- recurrent depressive disorder	−11.21				
-- bipolar affective disorder	−9.69				
Factors influencing health status and contact with health services	127.89	0.0002	0.0004	1.7428	0.11
-- contact with and exposure to communicable diseases	153.92				
-- occupational exposure to risk factors	6.99				
-- examination or observation for other reasons	296.96				
-- problems related to other psychosocial circumstances	1243.62				
-- general examination or investigation of persons without complaint or reported diagnosis	356.79				
Diseases of the nervous system	−43.08	0.0002	0.0001	0.6640	0.14
-- mononeuropathies of upper limb	−43.31				
-- epilepsy	−60.60				
-- nerve root or plexus disorders	−42.66				
-- mononeuropathies of lower limb	−57.16				
-- nerve root and plexus compressions in diseases classified elsewhere	169.49				

The items grouped under injury, poisoning and certain other consequences of external causes had the highest incidence rates, especially injuries to the wrist or hand, which was also the type of injury that was most common compared to the other specific causes (Additional file 1: Table S1). The second most common group of causes of work-related accidents was diseases of the musculoskeletal system and connective tissue, especially work-related back pain (Additional file 1: Table S1). External causes of morbidity and mortality were the third highest group of causes of work-related accidents, featuring being bitten or struck by a dog (Additional file 1: Table S1). Mental and behavioral disorders took fourth place, especially reactions to severe stress and adjustment disorders (Additional file 1: Table S1). In fifth place came factors influencing health status and contact with health services, the highest of which was due to contact with and exposure to communicable diseases (Additional file 1: Table S1). This was the group of causes of work-related accidents that saw the highest increase in the incidence of work-related injury/ill-health (Table 1). The lowest incidence rates of work-related accident/ill-health were found for diseases of the nervous system, where the main causes were mononeuropathies of an upper limb (Additional file 1: Table S1). This group of causes of work-related accidents, together with diseases of the musculoskeletal system and connective tissue, saw the greatest reductions in the rates of work-related accidents over the period studied (Table 1).

As for the specific causes of accidents/ill-health, the highest reductions came in synovitis and tenosynovitis; epilepsy; mononeuropathies of a lower limb; back pain; assault by being cut or pierced; and depressive episodes (Table 1). On the other hand, some causes increased, specifically problems related to other psychosocial circumstances; general examination or investigation of persons without complaint or reported diagnosis; examination or observation for other reasons; nerve root and plexus compressions in diseases classified elsewhere; and contact with and exposure to communicable diseases (Table 1).

A comparison of the accumulated incidence rates before and after the introduction of the Accident Prevention Factor revealed a drop in the following groups of causes of work-related accidents: injury, poisoning and certain other consequences of external causes; diseases of the musculoskeletal system and connective tissue; mental and behavioral disorders; and diseases of the nervous system (Table 1). In the same comparison, an increase was found in the groups of external causes of morbidity and mortality and factors influencing health status and contact with health services (Table 1). No statistical difference was identified when it came to the distribution of the accidents in the groups classified according to the causes of work-related accidents (*p* > 0.05).

### Economic activities

As for the economic activities involved, a reduction in work-related accidents and ill-health was found across all the groups studied: manufacturing and production; retail and motor vehicle repair; human health and social services; civil construction; and transportation, storage, and mail services (Table 2). High incidence rates were found in manufacturing and production, especially jobs involved in the slaughter of four-footed livestock (except pigs) (Additional file 2: Table S2). High rates were also found in human health and social services, especially hospital care (Additional file 2: Table S2); indeed, this group of economic activities showed the lowest percentage drop in work-related accidents/ill-health (Table 2). The third highest rate of work-related accidents and ill-health was found in transportation, storage, and mail services, with mail services being the area associated with the highest rates in this group (Additional file 2: Table S2). The construction sector was the group of economic activities that had the fourth highest rate of work-related accidents and ill-health, with the highest values being encountered in construction work in power generation and distribution and telecommunications (Additional file 2: Table S2). The group of economic activities with the lowest rates was retail and motor vehicle repair, in which the highest incidences were for beverage wholesale activities (Additional file 2: Table S2).

**Table 2:** Incidence of work-related accidents/ill-health in Brazil per economic activity (per 1000 workers)

Occupation	Difference in incidence between 2008 and 2014 (%)	Accumulated Incidence 2008–2009	Accumulated Incidence 2011–2014	Ratio of Accumulated Incidence Rates	Mann-Whitney Test ***p***
Manufacturing and production	−39.59	0.0370	0.0277	0.7475	0.005
-- Unrefined sugar production	− 58.80				
-- Slaughter of pigs, poultry, and other small livestock	−32.74				
-- Manufacture of parts and accessories for automotive vehicles not specified elsewhere	− 51.86				
-- Slaughter of four-footed livestock, except pigs	−46.27				
-- Manufacture of clothing, except underwear	− 68.75				
Retail and motor vehicle repair	−38.31	0.0130	0.0101	0.7782	0.15
-- Retail of goods in general, especially foodstuffs – hypermarkets and supermarkets	−34.24				
-- Retail of hardware, timber, and construction materials	− 41.88				
-- Trade in parts and accessories for motor vehicles	−41.35				
-- Retail in other new products not specified elsewhere	−38.83				
-- Wholesale of beverages	−40.19				
Human health and social services	−11.67	0.0370	0.0345	0.9340	0.62
-- Hospital care activities	−11.75				
-- Auxiliary diagnostics and therapeutic services	−29.11				
-- Outpatient medical and dental care	62.66				
-- Health care services not specified elsewhere	11.96				
-- Community care services	−19.15				
Civil construction	− 36.89	0.0257	0.0197	0.7657	0.12
-- Building construction	−43.55				
-- Construction work in power generation and distribution and telecommunications	12.43				
-- Road and railroad building	−32.39				
-- Civil construction not specified elsewhere	−39.72				
-- Real estate development	−12.41				
Transportation, storage, and mail services	−34.86	0.0279	0.0221	0.7944	0.40
-- Road freight transportation	−45.55				
-- Mail services	18.86				
-- Inter-municipal, inter-state, and international mass passenger road transportation with fixed itineraries	− 52.34				
-- Storage	−36.67				
-- Municipal and metropolitan mass passenger road transportation with fixed itineraries	−58.66				

If we analyze the most prevalent activities – the ones that were most prevalent in each group of economic activities – we find that the ones with the biggest drops in accident/illness rates were clothing manufacture (except underwear), unrefined sugar production, and municipal and metropolitan mass passenger road transportation with fixed itineraries (Table 2). Meanwhile, the individual economic activities where accident/ill-health rates rose were: outpatient medical and dental care, mail services, construction work in power generation and distribution and telecommunications, and health care services not specified elsewhere (Table 2).

When the incidence rates were analyzed in the groups of economic activities, before and after the introduction of the Accident Prevention Factor, no statistically significant difference was found in the distribution of the work-related accidents/ill-health (*p* > 0.05) except in the group of economic activities called manufacturing and production (Table 2).

### Severity of the accidents

The incidence rates were also analyzed according to the different levels of severity (Table 3). A reduction was found in the incidence in all the categories of severity, with the biggest reduction being found in accidents/ill-health resulting in over 15 days off work.

**Table 3:** Incidence of work-related accidents/ill-health in Brazil according to their severity (per 1000 workers)

Consequence	2008	2009	2010	2011	2012	2013	2014	Difference between 2008 and 2014 (%)
Medical attention	2.67	2.50	2.20	2.19	2.30	2.24	2.16	−19.14
Less than 15 days off work	8.06	7.45	6.81	6.69	6.69	6.85	7.01	−13.02
More than 15 days off work	8.51	7.89	6.95	6.52	6.07	5.75	5.08	−40.35
Permanent disability	0.33	0.35	0.32	0.32	0.36	0.35	0.28	−15.96
Death	0.07	0.06	0.06	0.06	0.06	0.06	0.06	−21.40

## Discussion

The reduction in the incidence of work-related accidents and ill-health in Brazil in recent years may be related to public policies designed to protect workers’ health and safety [13–15]. However, the reduction was not homogeneous across all the main causes of accidents and ill-health. A larger reduction can be seen in the following groups of causes of work-related accidents: injury, poisoning and certain other consequences of external causes; diseases of the musculoskeletal system and connective tissue; mental and behavioral disorders; and diseases of the nervous system.

The group of causes of accidents with the highest incidence rate over the period in question was injury, poisoning and certain other consequences of external causes. These include traffic accidents involving people engaged in work activities, as well as assaults and attacks, reflecting the overall upsurge in urban violence [16].

Back pain was the most common item in the group of work-related accidents/illness associated to diseases of the musculoskeletal system and connective tissue. This indicates the existence of a serious public health issue caused by this kind of ill-health, which can impair the well-being of the economically active population at some time in their lives [17]. It could be said that the policies that deal with workplace ergonomics have not proved effective in bringing about the desired changes. That is why it is important for new strategies to be developed that focus on the organization of work processes and technology infrastructure to bring about a reduction in the loads that are associated with back pain [18]. Meanwhile, work-related synovitis and tenosynovitis was found to be common. Although their incidence dropped in the period under study, this could be associated with underreporting [19].

When the causes of the work-related accidents related to mental and behavioral disorders were analyzed, a reduction in their incidence was found. This is inconsistent with what has been reported in the literature – a rising trend in the incidence of mental disorders [20, 21]. This reduction could be because Brazilian legislation already addresses subjective considerations in the analysis of the work environment [22]. It should be pointed out that in Brazil, the national occupational health care network (RENAST) has invested heavily in work-related mental health surveillance by shifting the focus of attention from intervention to the active role of workers, now seen as key players in addressing the challenges in this field [23].

It should also be noted that mental disorders could be under-recognized by workers themselves and by the persons responsible for occupational health. This could result in the underreporting of cases, giving a false impression that the incidence went down.

Nonetheless, satisfactory results were not found across all types of mental and behavioral disorders, particularly reactions to severe stress and adjustment disorders and other anxiety disorders (Table 1 and Additional file 1: Table S1 ). We would recommend that studies be done to identify the associated factors so that strategies can be devised to help reduce such cases.

As for the incidence of work-related ill-health affecting the nervous system, the most common are mononeuropathies of an upper limb. This finding is consistent with the literature [24]. Interestingly, the increased incidence of nerve root and plexus compressions in diseases classified elsewhere could be related to the introduction of a new method in 2007 by the social security system designed to identify which diseases and accidents are related to which kinds of occupations, thereby helping to improve the identification and notification of such cases [25].

An increase was observed in the incidence rates of external causes of morbidity and mortality and factors influencing health status and contact with health services.

Among the external causes of morbidity and mortality, the biggest cause of work-related accidents was being bitten or struck by a dog. This kind of accident is common amongst workers who work in public places, such as mail carriers, community health agents, etc., demonstrating the need for preventive measures for these kinds of activities [26, 27].

Work-related accidents relating to factors influencing health status and contact with health services saw the highest growth. This was found for all the specific causes contained in this group (factors influencing health status and contact with health services). Constant exposure to biological risks due to contact with bodily secretions and the handling of potentially contaminated material could play a part in this trend. Increasingly inadequate work environments associated with the fear of contamination arising from accidents mean that the reporting of this kind of accident is more consistent [6]. The increased incidence of accidents taking place during outpatient medical and dental care seems to be related to this. Another question observed in this group (factors influencing health status and contact with health services) was the increased incidence of problems related to other psychosocial circumstances. Failure to observe standard procedures to prevent psychosocial risks in the workplace could have contributed to the increased incidence [28].

The results demonstrate a higher frequency of accidents in manufacturing and production, corroborating the findings of other authors [29, 30]. Financial incentives offered to workers to increase their productivity run directly counter to accident prevention policies. This is borne out in the high rates of accidents in industrial activities [31]. However, manufacturing and production were also the group of economic activities with the greatest reduction in work-related accidents/ill-health.

In manufacturing and production, one of the workplaces where most accidents occur is at abattoirs. This could have to do with the working conditions and work relations in this sector, indicating the need to make consistent improvements to workers’ health and safety [32].

The group of economic activities with the highest recorded rates of occupational accidents and ill-health was human health and social services, topped by hospital care. High levels of stress are common in such activities, related to emotional ties to patients and their relatives, working night shifts, working more than one job, and working excessively long hours [33–35]. These factors not only increase the likelihood of accidents, but also impair workers’ quality of life [36–38]. Jobs involving the provision of human health care could be improved if actions included more effective standard practices for accident/disease prevention and if these were designed with the active participation of the workers in question [39].

In transportation, storage, and mail services, mail services were the economic activity group with the highest rates and an upward trend in the period under study. Adequate preventive policies and measures are needed to deal with this kind of activity, which is often undertaken in unfavorable social and environmental conditions and involves repetitive solitary actions over long periods of time, increasing the likelihood of accidents [27].

In the area of civil construction, the high and growing incidence of accidents in the areas of power generation and distribution and telecommunications indicate a reality that needs to be addressed. The outsourcing of construction work for big projects of this kind has contributed to a rationale whereby the employer gains financially, while worker safety issues are underplayed [40]. Also, construction work in power generation and distribution and telecommunications is associated with the most serious accidents, resulting in death, pointing to the need for greater attention on the part of government inspectors in these areas [41].

In retail and motor vehicle repair, the highest accident/ill-health rates were found in workers engaged in the wholesale of beverages. According to the literature, this is an activity that involves the transportation of goods. The high rates call not just for preventive actions for workers’ health, but also inter-sectoral policies to help prevent traffic accidents [42].

In view of the limitations of the method used in this study, it would be worthwhile doing new research to ascertain what the most frequent causes of the work-related accidents in each economic activity are. This information could be used by policymakers to improve occupational accident prevention policies in a more targeted manner.

If we compare the rates of work-related accidents before and after the introduction of the Accident Prevention Factor, the only significant difference can be seen in manufacturing and production. As our results demonstrate, preventable work-related accidents continue to be commonplace in Brazil. It would therefore be fair to say that Brazil has failed to keep pace with the reduction in work-related accidents witnessed in industrialized countries, brought about by improving preventive practices and introducing structural changes to the work environment [3]. This suggests investments are still required to reduce work-related accidents in most sectors of the Brazilian economy, including in manufacturing and production, which, despite achieving a significant reduction, still report work-related accidents and still have further to go to provide safe and healthy working environments.

One way accident rates could be improved, as reported in the literature, would be to bring about increased integration between the stakeholders, including employers, workers, and government institutions [43].

It should be pointed out that the Accident Prevention Factor targets the frequency and severity of accidents, but does not discuss issues intrinsically related to occupational health and safety, which could lead to the underreporting of accidents and limitations in tackling specific preventive measures in certain occupations [14, 44]. Furthermore, investigations of work-related accidents in Brazil are conducted using a variety of methods. The quality of these investigations needs to be improved, with the adoption of standard forms and procedures for recording accidents so as to build up a better picture of how they happen and thus to plan more effective prevention measures, resulting in reduced incidence rates [45, 46].

As for the severity of the accidents and illnesses, an overall reduction was observed. In the figures on accidents that require medical attention, underreporting could be at play, with employers choosing not to report minor injuries. Furthermore, since the Accident Prevention Factors means they are liable to pay higher taxes if a workplace injury occurs, this may reinforce their inclination not to report [35].

When it comes to more serious accidents, including those resulting in death, underreporting is less likely given the broader repercussions and the investigations triggered in such cases [16, 33, 34]. We would therefore suggest that the Accident Prevention Factor has had the effect of reducing cases of more serious accidents because of the financial implications for employers. The decline observed in the incidence of accidents resulting in death is also supported by the literature [14].

The falling rates of work-related accidents/ill-health resulting in time off work and permanent disability are indicative of measures being taken to improve working conditions. However, more studies are needed to identify more precisely which components are responsible for bringing about this change [15].

### Limitations

The limitations of this study include the potential for underreporting of accidents given the composition of the database. Also, the time series was limited to 2008–2014. Data from before 2008 were not included because of the way work-related accidents/ill-health are reported, which changed in May 2007. Data from after 2014 had not yet been consolidated by the social security system when the data were collected for this study. Another limitation is that the database does not include people in informal employment and the analysis covered only accidents/ill-health in the groups associated with the highest accident/illness rates (both cause of accident/ill health and economic activity). In each group analyzed, the study also only presented the causes and economic activities with the highest incidence rates. As such, ongoing studies are recommended to get a better understanding of how work-related accidents and ill-health evolve over the years.

## Conclusion

In the period under study, the incidence of accidents and ill-health mostly diminished, except for those involving external causes of morbidity and mortality and factors influencing health status and contact with health services. In the analysis, the severity of the accidents/ill-health was found to be on the decline in all the categories evaluated. However, a significant reduction in work-related accidents after the introduction of the Accident Prevention Factor was only found for manufacturing and production activities. This could well be related to the fact that this policy is based on broad principles that do not take into account particularities linked to each type of cause and economic activity in each working environment. In this respect, the policy could be improved. Progress could be made in the quality of accident investigations, in the way accidents are recorded and analyzed, and in identifying the specific root causes behind each activity so as to pinpoint actions according to each causal agent. This could help reduce accidents and ill-health in general.

## Supplementary Information

**Additional file 1: Table S1.** Incidence of work-related accidents/ill-health in Brazil using the ICD-10 (per 1000 workers), 2008 to 2014.

**Additional file 2: Table S2.** Incidence of work-related accidents/ill-health in Brazil per economic activity (per 1000 workers), 2008 to 2014.

## Data Availability

The datasets generated and analyzed during the current study are publicly available in the Brazilian government’s social security platform: https://www3.dataprev.gov.br/aet/ and statistical yearbooks (https://www.previdencia.gov.br/dados-abertos/dados-abertos-sst).

## Abbreviations

ICD-10: International Statistical Classification of Diseases
CNAE 2.0: Classificação Nacional de Atividades Econômicas
SPSS: Statistical Package for the Social Science

## References

[1] Budd JW, Spencer DA (2015). Worker well-being and the importance of work: bridging the gap. Eur J Ind Relat.

[2] Cabral LAA, Soler ZASG, Lopes JC (2014). "dual causation accident": a third type of work-related accident and its importance for occupational health surveillance. Cienc Saude Coletiva.

[3] Takala J, Hämäläinen P, Saarela KL, Yun LY, Manickam K, Jin TW, et al. Global estimates of the burden of injury and illness at work in 2012. J Occup Environ Hyg. 2014;11(5):326–37. 10.1080/15459624.2013.863131. 10.1080/15459624.2013.863131 PMC4003859 24219404

[4] Brasil. Ministério da Previdência Social. Bases de dados históricos da Previdência Social. 2017. http://www3.dataprev.gov.br/temp/DACT01consulta32082337.htm. Accessed 4 Oct 2017.

[5] Brasil. Plano Nacional de Segurança e Saúde no Trabalho. 2012. http://www.anamt.org.br/site/upload_arquivos/arquivos_diversos_2102014153407055475.pdf. Accessed 4 Oct 2012.

[6] Oliveira AC, Paiva MHRS (2013). Analysis of occupational accidents with biological material among professionals in pre-hospital services. Rev Lat-Am Enferm.

[7] Vieira MSC. Uso da metodologia de relacionamento de bases de dados para qualificação da informação sobre os acidentes e agravos relacionados ao trabalho. 2014. https://docplayer.com.br/83645456-Uso-da-metodologia-de-relacionamento-de-base-de-dados-para-a-qualificacao-da-informacao-sobre-os-acidentes-e-agravos-relacionados-ao-trabalho.html. .

[8] Rios MA, Nery AA, Rios PAA, Casotti CA, Cardoso JP (2015). Factors associated with work-related accidents in the informal commercial sector. Cad Saude Publica..

[9] Chávez S, Altman CE (2017). Gambling with life: masculinity, risk, and danger in the lives of unauthorized migrant roofers. A J Ind Med.

[10] Rigon VR, Turina AO (2017). A modernização das relações de trabalho e seus impactos previdenciários: o trabalho intermitente e o cálculo do índice FAP. Revista Ltr: legislação do trabalho.

[11] Brasil. Ministério da Previdência Social. www3.dataprev.gov.br/AEAT/greg/reg05/reg05.PHP. Accessed 4 Oct 2017.

[12] Brasil. Ministério da Previdência Social. Anuário Estatístico de Acidentes do Trabalho. www.previdencia.gov.br/dados-abertos/dados-abertos-sst. Accessed 4 Oct 2017.

[13] Alves MMM, Nomellini PF, Pranchevicius MCS (2013). Occupational mortality in Tocantins state, Brazil: a descriptive study 2000-2010. Epidemiol Serv Saude.

[14] Yoon SJ, Lin HK, Chen G, Yi S, Choi J, Rui Z (2013). Effect of occupational health and safety management system on work-related accident rate and differences of occupational health and safety management system awareness between managers in South Korea's construction industry. Saf Health Work.

[15] Almeida FSS, Morrone LC, Ribeiro KB (2014). Trends in incidence and mortality due to occupational accidents in Brazil, 1998-2008. Cad Saude Publica..

[16] Lacerda KM, Fernandes RCP, Nobre LCC, Pena PGL (2014). The (in) visibility of the fatal work-related injury as an external cause of accidents: a qualitative study. Rev Bras Saude Ocup..

[17] Medeiros JD, Pinto APS (2014). Impacto social e econômico na qualidade de vida dos indivíduos com lombalgia: revisão sistemática. Caderno de Graduação Ciências Biológicas e da Saúde – UNIT ALAGOAS.

[18] Meucci RD, Fassa AG, Faria NM (2015). Prevalence of chronic low back pain: systematic review. Rev Saude Publica.

[19] Andrade DM, Barbosa-Branco A (2015). Synovitis and tenosynovitis in Brazil: analysis of sickness benefit claims. Rev Bras Epidemiol.

[20] Carrillo-Castrillo JA, Rubio-Romero JC, Guadix J, Onieva L (2016). Identification of areas of intervention for public safety policies using multiple correspondence analysis. Dyna..

[21] Holden L, Scuffham PA, Hilton MF, Ware RS, Vecchio N, Whiteford HA (2011). Health-related productivity losses increase when the health condition is co-morbid with psychological distress: findings from a large cross-sectional sample of working Australians. BMC Public Health.

[22] Matos AB, Hostensky EL (2016). Preventive accident factor (PAF) and social security technical-epidemiological Nexus (SSTEN): indicators for a psychosocial intervention. Psicol Soc.

[23] Araújo TM, Palma TF, Araújo NC. Work-related Mental Health Surveillance in Brazil: characteristics, difficulties, and challenges. Cienc Saude Coletiva. 2017. 10.1590/1413-812320172210.17552017. 10.1590/1413-812320172210.17552017 29069180

[24] Oliveira Filho JD, Silva VTO, Dantas RA, Assis TO. Efeitos da mobilização neural na reabilitação de portadores de bursite crônica ocupacional no ombro. 2017. http://www.editorarealize.com.br/revistas/conbracis/trabalhos/TRABALHO_EV071_MD4_SA9_ID1725_02052017132303.pdf. Accessed 01 Dec 2018.

[25] Nasrala Neto E, Bittencourt WS, Nasrala MLS, Sousa FP, Roder IB (2014). The influence of Brazilian law on the notification of repetitive strain injury (RSI) and work-related musculoskeletal disorders (WMSDs). UNOPAR Cient Cienc Biol Saude.

[26] Almeida MCS (2013). Acidentes de trabalho ocorridos com agentes comunitários de saúde no Município de Caraguatatuba-SP.

[27] Mascarenhas FAN, Barbosa-Branco A (2014). Work-related disability among postal employees: incidence, duration, and social security costs in 2008. Cad Saude Publica..

[28] Ansoleaga E, Garrido P, Domínguez C, Castillo S, Lucero C, Tomicic A, et al. Facilitadores del reintegro laboral en trabajadores con patología mental de origen laboral: una revisión sistemática. Rev Med Chile. 2015;143(1):85–95. 10.4067/S0034-98872015000100011. 10.4067/S0034-98872015000100011 25860273

[29] Vasconcellos MC, Pignatti MG, Pignati WA (2009). Employment and occupational accidents in the slaughterhouse industry in expansion areas of agribusiness, Mato Grosso. Brasil Saude Soc.

[30] McLeod C, Sarkany D, Davies H, Lyons K, Koehoorn M (2015). Prevention in dangerous industries: does safety certification prevent tree-faller injuries?. Scand J Work Env Hea.

[31] Pouliakas K, Theodossiou I (2013). The economics of health and safety at work: an interdiciplinary review of the theory and policy. J Econ Surv.

[32] Jakobi HR, Barbosa-Branco A, Bueno LF, Ferreira RGM, Camargo LMA (2015). Sick leave benefits for workers in the Brazilian meat and fish industries in 2008. Cad Saude Publica.

[33] Fernandes JS, Miranzi SSC, Iwamoto HH, Tavares DMS, Santos CB (2012). A relação dos aspectos profissionais na qualidade de vida dos enfermeiros das equipes Saúde da Família. Rev Esc Enferm USP.

[34] Bracarense CF, Costa NS, Duarte JMG, Ferreira MBG, Simões ALA (2015). Quality of life at work: speech of professionals of the family health strategy. Esc Anna Nery.

[35] Silva MA, Lampert SS, Bandeira DR, Bosa CA, Barroso SM (2017). Emotional health of community agents: burnout, stress, well-being and quality of life. Rev SPAGESP.

[36] Amoafo E, Hanbali N, Patel A, Singh P (2014). What are the significant factors associated with burnout in doctors?. Occup Med-C.

[37] Chau YM, West S, Mapedzahama V (2014). Night work and the reproductive health of women: an integrated literature review. J Midwifery Wom Heal.

[38] Teles MAB, Barbosa MR, Vargas AMD, Gomes VE, Ferreira EF, AMEBL M, et al. Psychosocial work conditions and quality of life among primary health care employees: a cross sectional study. Health Qual Life Out. 2014;12:72. 10.1186/1477-7525-12-72 PMC4122097 24884707

[39] Costa D, Lacaz FAC, Jackson Filho JM, Vilela RAG (2013). Worker’s health within the Brazilian unified health system: challenges for a public policy. Rev Bras Saude Ocup.

[40] Filgueiras VA (2014). Terceirização e os limites da relação de emprego: trabalhadores mais próximos da escravidão e morte.

[41] Vasconcelos FD (2014). Brazilian Ministry of Labor’s inspection on workers’ safety and health, Brazil, 1996-2012. Rev Bras Saude Ocup..

[42] Silva W (2017). Análise da distribuição geográfica dos acidentes de trânsito na cidade de Natal, utilizando o método de Kernel.

[43] Morillas RM, Rubio-Romero JC, Fuertes A (2013). A comparative analysis of occupational health and safety risk prevention practices in Sweden and Spain. J Saf Res.

[44] Shur PZ, Zaĭtseva NV, Alekseev VB, Shliapnikov DM (2015). Occupational health risk assessment and management in workers in improvement of national policy in occupational hygiene and safety. Gig Sanit.

[45] Jacinto C, Soares CG, Fialho T, Antão P, Silva SA (2011). An overview of occupational accidents notification systems within the enlarged EU. Work..

[46] Salguero-Caparros F, Suarez-Cebador M, Carrillo-Castrillo JA, Rubio-Romero JC (2018). Quality evaluation of official accident reports conducted by labour authorities in Andalusia (Spain). Work..

