# Supplementary material for: Analysis of work-related accidents and ill-health in Brazil since the introduction of the accident prevention factor
Source: BMC Public Health. 2021 Apr 14;21:725. doi: 10.1186/s12889-021-10706-y (PMC8048148; doi:10.1186/s12889-021-10706-y)
Supplement: Supplementary file 1 — Additional file 1: Table S1. Incidence of work-related accidents/ill-health in Brazil using the ICD-10 (per 1000 workers), 2008 to 2014. [file 12889_2021_10706_MOESM1_ESM.docx]

Table S1 Incidence of work-related accidents/ill-health in Brazil using the ICD-10 (per 1000 workers), 2008 to 2014

| **Causes of accidents/ill-health** | **2008** | **2009** | **2010** | **2011** | **2012** | **2013** | **2014** |
| --- | --- | --- | --- | --- | --- | --- | --- |
| Injury, poisoning and certain other consequences of external causes | 13.263 | 12.435 | 11.512 | 11.163 | 10.772 | 10.557 | 10.221 |
| -- injury to the wrist or hand | 2.061 | 1.872 | 1.667 | 1.565 | 1.468 | 1.411 | 1.372 |
| -- fracture at wrist or hand level | 1.241 | 1.147 | 1.139 | 1.097 | 1.044 | 1.020 | 0.962 |
| -- contusion of wrist or hand | 0.910 | 0.839 | 0.763 | 0.747 | 0.717 | 0.710 | 0.678 |
| -- dislocation or strain or sprain of joints or ligaments at ankle and foot level | 0.656 | 0.646 | 0.601 | 0.607 | 0.610 | 0.607 | 0.611 |
| -- fracture of leg, including ankle | 0.555 | 0.553 | 0.549 | 0.539 | 0.525 | 0.514 | 0.500 |
|  |  |  |  |  |  |  |  |
| Diseases of the musculoskeletal system and connective tissue | 3.630 | 3.144 | 2.630 | 2.436 | 2.247 | 2.180 | 2.046 |
| -- back pain | 1.422 | 1.154 | 0.932 | 0.842 | 0.762 | 0.712 | 0.665 |
| -- shoulder lesions | 0.599 | 0.528 | 0.465 | 0.456 | 0.434 | 0.450 | 0.424 |
| -- synovitis and tenosynovitis | 0.586 | 0.463 | 0.375 | 0.326 | 0.284 | 0.256 | 0.230 |
| -- other intervertebral disc injury | 0.180 | 0.193 | 0.155 | 0.147 | 0.135 | 0.132 | 0.121 |
| -- other joint derangements | 0.146 | 0.142 | 0.129 | 0.126 | 0.131 | 0.142 | 0.143 |
|  |  |  |  |  |  |  |  |
| External causes of morbidity and mortality | 0.449 | 0.445 | 0.423 | 0.426 | 0.453 | 0.473 | 0.476 |
| -- bitten or struck by dog | 0.033 | 0.033 | 0.034 | 0.035 | 0.037 | 0.038 | 0.036 |
| -- unintentional cut, puncture, perforation, or hemorrhage during medical or surgical procedure | 0.029 | 0.028 | 0.027 | 0.028 | 0.028 | 0.027 | 0.024 |
| -- unspecified harm during medical or surgical procedure | 0.017 | 0.017 | 0.019 | 0.019 | 0.018 | 0.022 | 0.022 |
| -- cut or piercing with a sharp object, intentionality not determined | 0.016 | 0.017 | 0.019 | 0.016 | 0.021 | 0.019 | 0.020 |
| -- assault by being cut or pierced | 0.021 | 0.022 | 0.017 | 0.015 | 0.017 | 0.014 | 0.011 |
|  |  |  |  |  |  |  |  |
| Mental and behavioral disorders | 0.445 | 0.424 | 0.357 | 0.352 | 0.363 | 0.395 | 0.385 |
| -- reactions to severe stress and adjustment disorders | 0.180 | 0.156 | 0.136 | 0.142 | 0.166 | 0.186 | 0.186 |
| -- depressive episodes | 0.132 | 0.119 | 0.093 | 0.087 | 0.076 | 0.080 | 0.074 |
| -- other anxiety disorders | 0.049 | 0.060 | 0.054 | 0.055 | 0.058 | 0.065 | 0.066 |
| -- recurrent depressive disorder | 0.025 | 0.029 | 0.024 | 0.022 | 0.022 | 0.023 | 0.023 |
| -- bipolar affective disorder | 0.011 | 0.014 | 0.013 | 0.011 | 0.010 | 0.011 | 0.010 |
|  |  |  |  |  |  |  |  |
| Factors influencing health status and contact with health services | 0.216 | 0.258 | 0.276 | 0.333 | 0.394 | 0.432 | 0.493 |
| -- contact with and exposure to communicable diseases | 0.092 | 0.120 | 0.134 | 0.153 | 0.182 | 0.204 | 0.235 |
| -- occupational exposure to risk factors | 0.058 | 0.056 | 0.051 | 0.050 | 0.051 | 0.054 | 0.062 |
| -- examination or observation for other reasons | 0.014 | 0.019 | 0.023 | 0.028 | 0.035 | 0.042 | 0.055 |
| -- problems related to other psychosocial circumstances | 0.003 | 0.006 | 0.009 | 0.035 | 0.055 | 0.050 | 0.042 |
| -- general examination or investigation of persons without complaint or reported diagnosis | 0.008 | 0.011 | 0.011 | 0.015 | 0.020 | 0.027 | 0.036 |
|  |  |  |  |  |  |  |  |
| Diseases of the nervous system | 0.251 | 0.214 | 0.180 | 0.167 | 0.154 | 0.154 | 0.143 |
| -- mononeuropathies of upper limb | 0.219 | 0.185 | 0.154 | 0.142 | 0.132 | 0.133 | 0.124 |
| -- epilepsy | 0.005 | 0.005 | 0.004 | 0.004 | 0.003 | 0.003 | 0.002 |
| -- nerve root or plexus disorders | 0.005 | 0.004 | 0.004 | 0.004 | 0.003 | 0.003 | 0.003 |
| -- mononeuropathies of lower limb | 0.004 | 0.004 | 0.003 | 0.002 | 0.002 | 0.002 | 0.002 |
| -- nerve root and plexus compressions in diseases classified elsewhere | 0.001 | 0.001 | 0.002 | 0.002 | 0.003 | 0.003 | 0.002 |
