# Supplementary material for: Analysis of work-related accidents and ill-health in Brazil since the introduction of the accident prevention factor
Source: BMC Public Health. 2021 Apr 14;21:725. doi: 10.1186/s12889-021-10706-y (PMC8048148; doi:10.1186/s12889-021-10706-y)
Supplement: Supplementary file 2 — Additional file 2: Table S2. Incidence of work-related accidents/ill-health in Brazil per economic activity (per 1000 workers), 2008 to 2014. [file 12889_2021_10706_MOESM2_ESM.docx]

Table S2 – Incidence of work-related accidents/ill-health in Brazil per economic activity (per 1000 workers), 2008 to 2014

| **Occupation** | **2008** | **2009** | **2010** | **2011** | **2012** | **2013** | **2014** |
| --- | --- | --- | --- | --- | --- | --- | --- |
| Manufacturing and production | 39.29 | 34.87 | 30.91 | 30.09 | 28.64 | 28.40 | 23.74 |
| -- Unrefined sugar production | 72.63 | 62.47 | 54.27 | 47.67 | 43.49 | 36.73 | 29.92 |
| -- Slaughter of pigs, poultry, and other small livestock | 58.69 | 59.15 | 47.99 | 42.84 | 47.37 | 41.21 | 39.48 |
| -- Manufacture of parts and accessories for automotive vehicles not specified elsewhere | 59.34 | 46.60 | 39.46 | 38.59 | 34.84 | 34.85 | 28.57 |
| -- Slaughter of four-footed livestock, except pigs | 76.22 | 66.85 | 59.49 | 56.11 | 51.32 | 51.79 | 40.95 |
| -- Manufacture of clothing, except underwear | 19.29 | 19.16 | 13.65 | 12.66 | 10.56 | 10.58 | 6.03 |
|  |  |  |  |  |  |  |  |
| Retail and motor vehicle repair | 13.51 | 12.61 | 11.55 | 11.26 | 10.53 | 10.63 | 8.33 |
| -- Retail of goods in general, especially foodstuffs – hypermarkets and supermarkets | 23.60 | 22.97 | 20.98 | 20.95 | 19.35 | 19.34 | 15.52 |
| -- Retail of hardware, timber, and construction materials | 16.04 | 14.97 | 13.98 | 13.05 | 12.55 | 12.28 | 9.32 |
| -- Trade in parts and accessories for motor vehicles | 15.60 | 14.44 | 13.16 | 12.58 | 11.48 | 11.80 | 9.15 |
| -- Retail in other new products not specified elsewhere | 10.04 | 9.22 | 8.25 | 7.80 | 7.14 | 7.66 | 6.14 |
| -- Wholesale of beverages | 40.50 | 40.72 | 38.19 | 35.56 | 34.08 | 32.04 | 24.22 |
|  |  |  |  |  |  |  |  |
| Human health and social services | 36.76 | 37.27 | 35.98 | 35.68 | 35.04 | 35.42 | 32.47 |
| -- Hospital care activities | 54.79 | 56.19 | 54.45 | 53.64 | 53.28 | 52.85 | 48.35 |
| -- Auxiliary diagnostics and therapeutic services | 23.22 | 22.01 | 18.45 | 17.17 | 17.54 | 17.37 | 16.46 |
| -- Outpatient medical and dental care | 6.40 | 7.51 | 9.04 | 10.17 | 9.61 | 10.94 | 10.41 |
| -- Health care services not specified elsewhere | 20.94 | 19.92 | 21.15 | 21.04 | 19.87 | 23.42 | 23.45 |
| -- Community care services | 16.49 | 16.57 | 15.02 | 15.53 | 14.86 | 16.08 | 13.33 |
|  |  |  |  |  |  |  |  |
| Civil construction | 26.59 | 25.06 | 21.23 | 20.77 | 21.28 | 20.17 | 16.78 |
| -- Building construction | 24.62 | 22.63 | 18.96 | 19.46 | 19.60 | 18.81 | 13.90 |
| -- Construction work in power generation and distribution and telecommunications | 38.05 | 36.00 | 33.52 | 30.65 | 31.44 | 33.07 | 42.78 |
| -- Road and railroad building | 33.79 | 29.27 | 28.48 | 26.75 | 29.30 | 25.49 | 22.85 |
| -- Civil construction not specified elsewhere | 24.39 | 25.72 | 22.77 | 17.37 | 19.94 | 16.05 | 14.71 |
| -- Real estate development | 23.45 | 25.96 | 20.78 | 24.53 | 27.60 | 27.47 | 20.54 |
|  |  |  |  |  |  |  |  |
| Transportation, storage, and mail services | 28.50 | 27.37 | 24.83 | 23.53 | 23.51 | 23.38 | 18.57 |
| -- Road freight transportation | 27.06 | 25.68 | 22.94 | 21.30 | 20.59 | 19.83 | 14.73 |
| -- Mail services | 83.00 | 85.71 | 89.71 | 90.34 | 105.39 | 110.49 | 98.66 |
| -- Inter-municipal, inter-state, and international mass passenger road transportation with fixed itineraries | 70.34 | 69.88 | 58.99 | 55.80 | 51.97 | 52.20 | 33.53 |
| -- Storage | 31.59 | 31.10 | 28.41 | 25.58 | 23.47 | 22.20 | 20.00 |
| -- Municipal and metropolitan mass passenger road transportation with fixed itineraries | 5.33 | 5.08 | 4.20 | 3.93 | 3.51 | 3.62 | 2.20 |
